# Intraarticular osteotomy of malunited tibial plateau fractures: an analysis of clinical results with a mean follow-up after 4 years

**DOI:** 10.1007/s00068-020-01440-y

**Published:** 2020-07-25

**Authors:** Lena Alm, Jannik Frings, Matthias Krause, Karl-Heinz Frosch

**Affiliations:** 1 Department of Trauma and Orthopaedic Surgery, Sports Traumatology, BG Hospital Hamburg, Hamburg, Germany; 2 Department of Trauma and Orthopaedic Surgery, University Medical Center Hamburg-Eppendorf, Martinistraße 52, 20246 Hamburg, Germany

**Keywords:** Tibial plateau fracture, Malunion, Intraarticular osteotomy, Failure analysis

## Abstract

**Purpose:**

Malunions are a common complication after tibial plateau fractures (TPF), leading to stiffness, pseudo-instability and posttraumatic osteoarthritis. The purpose of this study was to analyse the clinical outcome after intraarticular osteotomy of malunited TPF and to perform a failure analysis.

**Methods:**

Between 2013 and 2018, 23 patients (11 males, 12 females; 43.8 ± 12.8 years) with intraarticular osteotomy after malunited TPF were included in the retrospective study. Clinical examination and postoperative scores were collected with a minimum follow-up of 24 months. Malunion was measured on pre- and postoperative CT scans and localized according to the 10-segment classification while the leg axis in the frontal plane was measured pre- and postoperatively on long leg standing radiographs.

**Results:**

Excellent and good clinical outcome was achieved in 73.9% (*n* = 17) of the cases and patient related outcome improved significantly (Tegner 3.3 ± 1.6–5 ± 1.8, *p* < 0.001; clinical Rasmussen 14.6 ± 3.8–24.9 ± 4.4, *p* < 0.001). Radiological parameters also improved as an intraarticular step-off was reduced from 9 ± 3.8 to 0.6 ± 0.8 mm (*p* < 0.001) and a lower limb malalignment from 7.2 ± 4.8° to 1.5 ± 1.9° (*p* = 0.003). Failure analysis showed that an impaired clinical result correlated with a postoperative extension (*n* = 3, *p* < 0.001) and flexion deficit (*n* = 4, *p* = 0.035).

**Conclusion:**

Intraarticular osteotomy of malunited TPF lead to good clinical results with significant clinical and radiological improvement in most cases while an impaired patient outcome correlated with a limited range of motion. This study is the first failure analysis of intraarticular osteotomy after malunited TPF published up to now.

## Introduction

The treatment of complex tibial plateau fractures (TPF) can be challenging, even for the experienced surgeon while complication rates can rise up to 40% [1, 2]. Limited range of motion, infection of the knee and malunion lead to impaired patient outcome after primary osteosynthesis [3, 4]. Malunion after primary osteosynthesis of the TPF often results in dysfunction of the knee as it can lead to chronic subluxation, pseudo-instability, stiffness and posttraumatic osteoarthritis [5, 6]. There are only a few treatment options, including the intraarticular osteotomy in order to improve malunion of the TPF [7, 8]. While intraarticular osteotomy of the malunited TPF is technical demanding, there is little evidence about the clinical outcome after surgical revision [5, 6].

The primary aim of this study was to present the outcome of patients treated with intra-articular osteotomy after malunited TPF according to the concept of the 10-segment classification [9, 10]. The secondary aim was to perform an analysis to point out risk factors for intraarticular osteotomy failure. We hypothesized that intraarticular osteotomy in malunited TPF can lead to good clinical results with significant radiological improvement while limited range of motion is associated with an impaired patient outcome.

## Patients and methods

### Study population

The retrospective cohort study took place at a level one trauma center from January 2013 to December 2018 and included all patients treated with intraarticular osteotomy after malunited TPF. Subsequently, 23 patients (43.8 ± 12.8 years) met the inclusion criteria of a minimum follow- up of 24 months. Exclusion criteria were the lack of pre- and postoperative computer tomography scans, age > 65 years, high degree of osteoarthritis (Kellgren & Lawrence grade 3 or 4) and isolated extra-articular fracture manifestation (OTA/AO type A).

The height of tibial plateau step-offs was measured on pre- and postoperative CT scans and localized according to the 10-segment classification while the leg axis (measured from the hip to the ankle) in the frontal plane was pre- and postoperatively measured on long leg standing radiographs [9, 11]. Conventional radiographs and CT scans were reviewed from the initial injury to the latest follow-up. Postoperative long leg standing radiographs were available for 56.5% of patients (*n* = 13).

In addition to postoperative clinical symptoms, the patient- related outcome was assessed by the functional scores (Visual analogue scale (VAS), Tegner and clinical Rasmussen pre- and postoperatively; Oxford Knee Score postoperatively) with a minimum follow-up of 24 months and patients were identified as “poor outcome” in case of TKA conversion or a poor patient reported outcome (Oxford Knee Score < 20 points) [12–15]. The study protocol was approved by the local ethics committee and an informed consent was obtained from all individual participants included in the study.

### Surgical technique

**Fig. 1:**
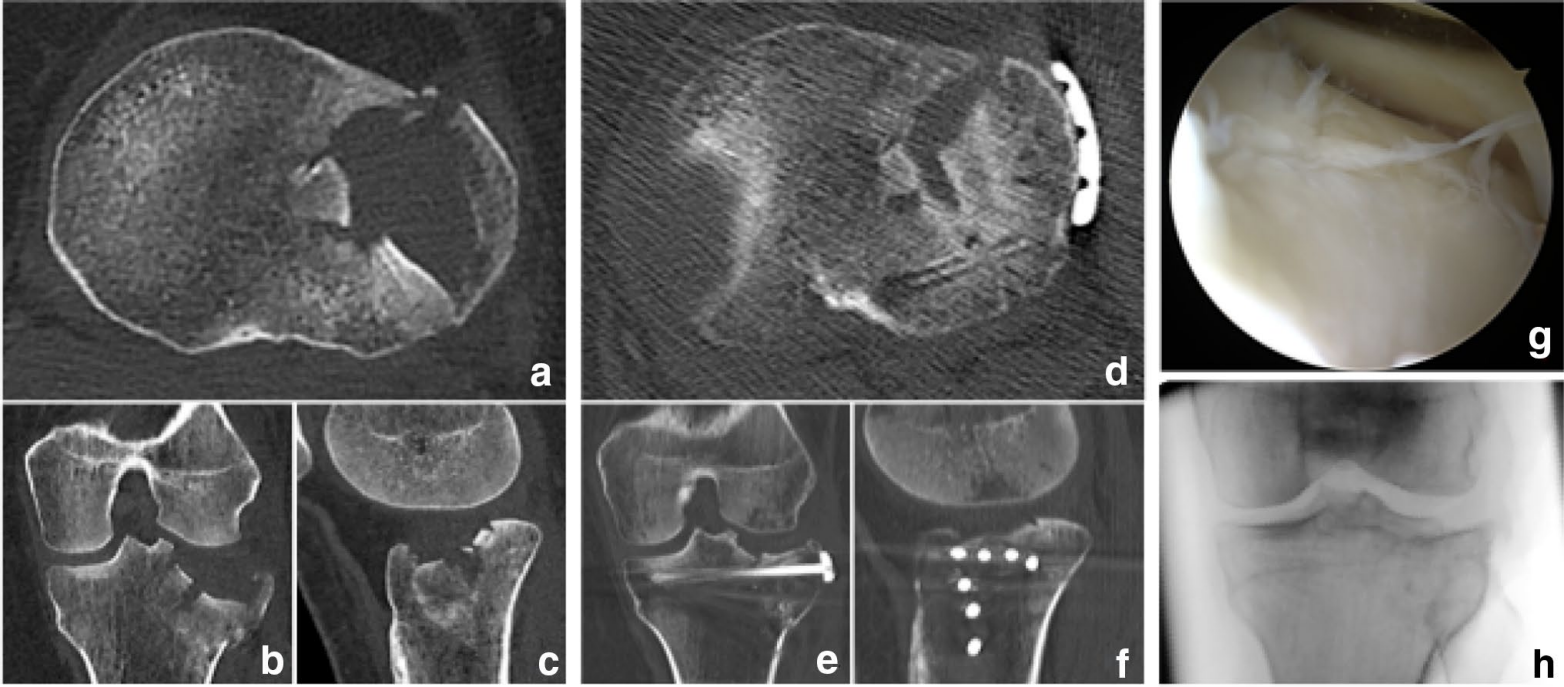
Primary osteosynthesis of an OTA/AO type B3 tibial plateau fracture (lateral split depression fracture, **a**–**c**) treated via an anterolateral approach and lateral plating (**d**–**f**). Due to a secondary loss of reduction of the lateral hemiplateau- also seen under arthroscopy and on fluoroscopy (**g**, **h**), the indication for an intraarticular osteotomy was placed (Fig. 2)

**Fig. 2:**
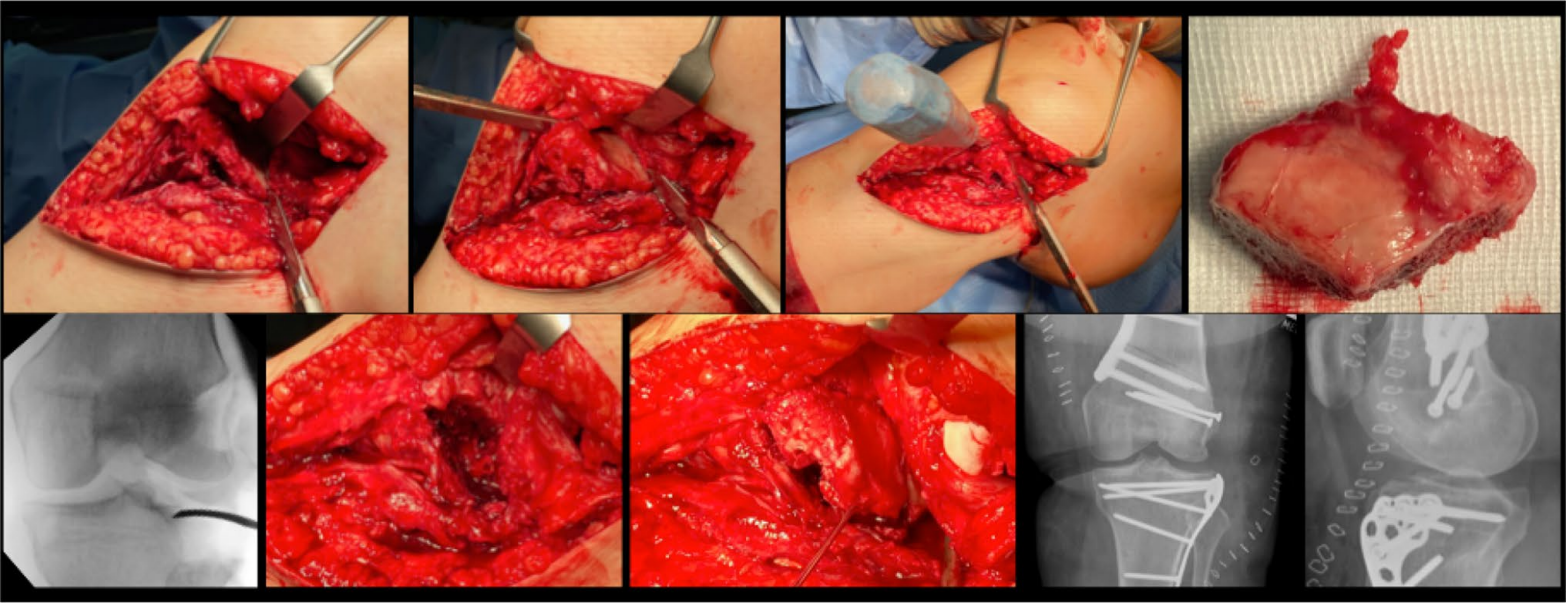
An anterolateral quadrant osteotomy via lateral extended approach (lateral epicondyle osteotomy) was performed in a patient showing an intraarticular step-off of the lateral hemiplateau after primary osteosynthesis of an OTA/AO B3 tibial plateau fracture (lateral split depression fracture, same patient as seen in Fig.1a-h). Anatomic articular reconstruction and distal femoral osteotomy was carried out in order to improve articular surface and leg alignment

All patients were classified according to the OTA/OA and 10-segment classification of the German Knee Society and the surgical approach and management of the malunited TPF was based on the concept of the 10-segment classification (Tables 1 and 2, Fig. 1) [16]. In general, a surgical revision was indicated in case of tibial plateau articular surface depression > 2 mm and mediolateral widening of the tibial plateau > 5 mm [8]. Depending on the malunion of the TPF, a segmental or quadrant osteotomy was performed (Table 3). Before addressing the malunited TPF, a diagnostic arthroscopy was carried out to evaluate possible concomitant lesions of the cartilage, meniscus or ligaments as well as to visualize the biomechanical impact of the malunion during joint movement. A lateral meniscus lesion that could not be repaired was a contraindication for a lateral or posterolateral segment or quadrant osteotomy- either visualized during arthroscopy or preoperative magnetic resonance imaging (MRI). Due to a lower complication profile compared to fibula osteotomy, a lateral epicondyle osteotomy (lateral extended approach, Fig. 2) was preferred in case of posterolateral malunited TPF with involvement of central segments [8]. After removal of the internal fixation of the primary osteosynthesis, the malunion was visualized and if necessary, callus was removed. An intraarticular osteotomy was placed at the height of the malunion and optionally underlayed with autologous or allogeneic cancellous bone (Fig. 1).

**Table 1:** 10-segment classification and OTA/OA of the primary tibial plateau fracture

	AO type B (*n* = 16)	AO type C (*n* = 7)
10-segments (*n*/%)		
AMM	3/37.5	5/62.5
AMC	3/30	7/70
PMM	3/37.5	5/62.5
PMC	2/22.2	7/77.8
AC	5/41.7	7/58.3
PC	4/36.4	7/63.6
ALL	12/70.6	5/29.4
ALC	12/63.2	7/36.8
PLL	12/66.7	6/33.3
PLC	13/65	7/35

*AMM* antero-medio-medial, *AMC* antero-medio-central, *PMM* postero-medio-medial, *PMC* postero-medio-central, *AC* antero-central, *PC* postero-central, *ALL* antero-latero-lateral, *ALC* antero-latero-central, *PLL* postero-latero-lateral, *PLC* postero-latero-central

**Table 2:** 10-segment classification of the malunion (before osteotomy)

10-segments (*n*/%)	Malunion (*n* = 23)
AMM	5/21.7
AMC	6/26.1
PMM	4/17.4
PMC	4/17.4
AC	5/21.7
PC	3/13
ALL	14/60.9
ALC	15/65.2
PLL	16/69.6
PLC	15/65.2

*AMM* antero-medio-medial, *AMC* antero-medio-central, *PMM* postero-medio-medial, *PMC* postero-medio-central, *AC* antero-central, *PC* postero-central, *ALL* antero-latero-lateral, *ALC* antero-latero-central, *PLL* postero- latero-lateral, *PLC* postero-latero-central

**Table 3:** Surgical approach and intraarticular osteotomy regards to treatment success and poor outcome

Characteristics	Treatment success (*n* = 19)	Poor outcome (*n* = 4)	*p* value
Surgical approach (*n*)			
Anterolateral	5	2	
Posterolateral	1	0	n.s
Anteromedial	5	0	
Combined anterolateral + anteromedial	1	0	
Combined anterolateral posterolateral	1	0	
Lateral extended approach	8	2	
Intraarticular osteotomy (*n*)			
ALL + ALC segments	1	2	
ALC + PLC segments	1	0	
PLL + PLC segments	2	1	
ALL + PLL + PLC segments	1	0	n.s
AMM + AMC + AC segments	1	0	
Lateral hemiplateau	9	1	
Medial hemiplateau	4	0	

*n.s.* not significant, *AMM* antero-medio-medial, *AMC* antero-medio-central, *AC* antero-central, *ALL* antero-latero-lateral, *ALC* antero-latero-central, *PLL* postero- latero-lateral, *PLC* postero-latero-central

Definite fixation of the intraarticular osteotomy was applied with screw fixation or combined with plate fixation. Surgical approaches are displayed in Table 3.

### Rehabilitation protocol

Postoperative management was chosen according to the type of TPF malunion and osteotomy. Passive range-of-motion (between 60° and 90° of knee flexion depending on the TPF malunion) and mobilization were started on the first postoperative day in most patients, depending on the soft tissue status and partial weight-bearing was recommended for 6−8 weeks.

### Clinical testing protocol after intraarticular osteotomy

At the latest follow-up pain, knee joint function, range-of-motion (range 0–140°) and postoperative complications were summarised and a limited range of motion was stated when an extension or flexion deficit > 5° occurred. Anterior instability was measured by the Lachman test and posterior instability by the posterior drawer test while collateral knee instability was also documented [17]. Patient reported outcome measures were collected in using VAS, Tegner and clinical Rasmussen score before and after revision surgery and Oxford Knee Score after surgery.

### Statistical analysis

Statistical analysis was performed using IBM^®^SPSS^®^Statistics (Version 22, SPSS Inc, Chicago, IL, USA). Mean ± standard deviation was used for continuous variables and calculation was based on two groups: treatment success and poor outcome at follow up. Mean differences between these two groups were calculated with unpaired Student’s *t*-test in case of parametric parameters. Chi-Squared test was performed to compare categorical parameters. A *p*-value less than 0.05 was considered significant.

## Results

Intraarticular osteotomy after malunited TPF was performed in 23 patients (males = 11, females = 12) with a mean follow-up of 50.0 ± 17.2 (24–80) months. The osteotomy was performed in average 8.6 ± 2.4 (6–18) months after primary osteosynthesis. Mean age at the time of revision surgery was 43.8 ± 12.8 (20–65) years and mean body mass index 25.7 ± 4.4 (18–65). According to the OTA/OA classification we evaluated 16 patients with malunited AO type B (B1 = 8, B2 = 2, B3 = 6) and 7 patients with malunited AO type C (C1 = 1, C3 = 6) fractures. A malunion of the TPF was located in 5 cases on the medial and in 18 cases on the lateral hemiplateau of the tibia. Data is summarized in Tables 1, 2, 3, 4.

**Table 4:** Treatment success and poor outcome according to patient and surgery related factors

Characteristics	Treatment success (*n* = 19)	Poor outcome (*n* = 4)	*p* value
Age [mean ± SD (range)]	43.8 ± 13 (18–65)	45 ± 8.4 (36–54)	n.s
Sex, female (*n*)	9	3	n.s
Time between primary and revision surgery [months, mean ± SD (range)]	7.9 ± 1.4 (6–11)	10.8 ± 4.9 (8–18)	n.s
BMI [kg/m^2^; mean ± SD (range)]	25.4 ± 4.3 (19–32)	27.3 ± 5.1 (23–33)	n.s
ATO/AO type C (*n*)	5	2	n.s
Varus malalignment (*n*)			
Preoperative	3	0	n.s
Postoperative	0	0	n.s
Valgus malalignment (*n*)			
Preoperative	9	2	n.s
Postoperative	1	0	n.s
Intraarticular step-off postoperative (*n*)			
1 mm	2	1	n.s
2 mm	3	1	

*n.s.* not significant, *SD* standard deviation, *BMI* body-mass-index

In general, patient related outcome including Tegner (3.3 ± 1.6–5 ± 1.8, *p* < 0.001) and clinical Rasmussen (14.6 ± 3.8–24.9 ± 4.4, *p* < 0.001) significantly  improved and Oxford Knee Score showed postoperative a mean of 36.1 ± 11.2 points. Pain was significantly reduced from 5.3 ± 3 points to 1.5 ± 2.1 (< 0.001) at the time of follow-up. Also, radiological parameters significantly improved as an intraarticular step-off was reduced from average 9 ± 3.8 to 0.6 ± 0.8 mm (*p* < 0.001) and the leg axis, measured on long leg standing radiographs, improved from average 7.2 ± 4.8° to 1.5 ± 1.9° (*p* = 0.003) after osteotomy (Table 5). Excellent clinical outcome was achieved in 47.8% (*n* = 11) of the cases, good clinical outcome in 26.1% (*n* = 6), moderate in 13% (*n* = 3) of the cases and poor in 13% (*n* = 3) of the cases according to the Oxford Knee Score.

**Table 5:** Patient characteristics on pre- and postoperative assessment of radiological and functional results

Characteristics	Mean ± SD (range)	*p* value
Intraarticular step-off (mm)		
Preoperative	9 ± 3.8 (3–20)	< 0.001
Postoperative	0.6 ± 0.8 (0–2)	
Leg axis (°)		
Preoperative	7.2 ± 4.8 (2–17)	0.003
Postoperative	1.5 ± 1.9 (0–7)	
VAS (points)		
Preoperative	5.3 ± 3 (0–9)	< 0.001
Postoperative	1.5 ± 2.1 (0–8)	
Tegner (points)		
Preoperative	3.3 ± 1.6 (1–7)	< 0.001
Postoperative	5 ± 1.8 (1–8)	
Clinical Rasmussen (points)		
Preoperative	14.6 ± 3.8 (7–22)	< 0.001
Postoperative	24.9 ± 4.4 (16–30)	
Oxford knee score (points)		
Postoperative	36.1 ± 11.2 (10–48)	–

*n.s.* not significant, *SD* standard deviation

A specific malunited segment in regards to the 10-segment classification, the surgical approach and the type of intraarticular osteotomy were not significantly associated with a poor outcome (Table 3). Moreover, the analysis did not show a correlation between certain factors like age, sex, lower leg malalignment, postoperative intraarticular step-off and poor outcome (Table 4). Nevertheless, the most involved segments in malunion were the antero-latero-central (ALC), postero-latero-central (PLC) and postero-latero-lateral (PLL) segments (Table 2).

Analysing the postoperative clinical assessment, it was demonstrated that poor outcome correlated with a postoperative extension (*n* = 3, *p* < 0.001) and flexion deficit (*n* = 4, *p* = 0.035) (Table 6). Overall, postoperative complications were shown in 13 patients (56.5%). A limited range of motion occurred in 12 patients, revision with slope-correction osteotomy in one patient, two had a postoperative superficial wound infection which could be successfully treated by surgery. Another two patients converted to total knee arthroplasty (TKA) after 8 and 17 months after intraarticular osteotomy.

**Table 6:** Treatment success and poor outcome regards to postoperative clinical findings

Characteristics	Treatment success (*n* = 19)	Poor outcome (*n* = 4)	*p* value
Extension deficit (*n*)	0	3	< .001
Flexion deficit (*n*)	8	4	0.035
Postoperative extension deficit [° in mean ± SD (range)]	1.3 ± 1.2 (0–3)	5.8 ± 3 (3–10)	< 0.001
Postoperative flexion deficit [° in mean ± SD (range)]	8.6 ± 9.8 (0–45)	23.8 ± 12.5 (10–45)	0.013
Positive Lachman (*n*)	1	1	n.s
Positive posterior drawer test (*n*)	3	0	n.s
Collateral knee instability (*n*)	6	2	n.s

*n.s.* not significant, *SD* standard deviation

## Discussion

The main findings of this study were that intraarticular osteotomy of malunited TPF lead to good clinical results with significant improvement in most patients while limited range of motion is a significant factor for poor outcome. More than 70% of patients showed good to excellent postoperative outcomes; clinical as well as radiological parameters significantly improved. This study demonstrated that intraarticular osteotomy according to the concept of the 10-segment classification improves patient outcome [9–11, 18, 19].

Studies evaluating the outcome of patients after malunion of the TPF are rare. Kerkhoffs et al. analysed 23 patients with combined intra-articular elevation and opening wedge varus osteotomy with lateral articular surface collapse and valgus malunion of the tibia after a mean of 13 years [7]. They showed that a correction of the intraarticular depression and valgus malalignment provides good results and that the anatomic leg axis was restored in all patients. In line with Krettek et al. and Liangjun et al., we believe that a malunited TPF is better treated with intraarticular osteotomy to gain an anatomical articular surface and alignment restoration [5, 20]. Liangjun et al. analysed 25 patients with malunited TPF while in 15 cases an intraarticular osteotomy was performed and satisfactory patient outcomes were achieved [5]. Wang et al. demonstrated 13 patients with posterolateral tibial plateau fracture malunion and intraarticular osteotomy at a mean follow-up of 20 months [6]. The articular step-off was corrected in all patients from 15.4 mm preoperatively to 3.3 mm at latest follow-up and patients achieved satisfactory functional outcome and knee stability.

Although a few studies reported on the outcome of intraarticular osteotomy of malunited TPF and showed an improvement in radiological and clinical outcome, none of them have performed an analysis of poor outcome after intraarticular osteotomy. Furthermore this is the largest study with intraarticular osteotomy after failed TPF.

While patient related factors like age, sex, body mass index and time between primary osteosynthesis and revision surgery did not show any influence on a poor outcome after intraarticular osteotomy, also radiologic factors like leg malalignment and intraarticular step-offs up to 2 mm did not correlate with an impaired patient outcome. Numerous studies analysing the outcome of TPF have identified that an incongruous step-off > 2 mm is associated with early osteoarthritis and poor clinical outcome [21]. As postoperative step-offs were only up to 2 mm in postoperative assessment in this study, a correlation between postoperative intraarticular step-off and osteotomy failure could not be detected. Nevertheless, in this study most patients undertook a revision surgery after primary osteosynthesis because of pain and poor clinical outcome while CT scans revealed an average of 9 mm step-off before intraarticular osteotomy. Thus, a correlation between a step-off > 2 mm and impaired patient outcome can also be assumed in this study.

The area of malunion, the choice of surgical approach and the type of osteotomy based on the concept of the 10-segment classification did not influence the outcome.

Nevertheless, we found that a postoperative limited range of motion is associated with an impaired patient outcome after intraarticular osteotomy. Thus, an extension and flexion deficit can lead to poor patient outcome. According to various studies reporting the outcome of primary osteosynthesis of TPF, limited range of motion is common and lead to poor clinical results [3, 22]. Thus, postoperative rehabilitation should be more in focus of primary and revision surgery of TPF.

Overall, this study revealed that good clinical results can be achieved by an intraarticular osteotomy and that preoperative clinical and radiological parameters can be successfully improved. However, this procedure remains complex and can cause complications. Although two patients converted to a total knee arthroplasty, we are convinced that a reconstructive, knee joint preserving technique can delay the necessity of joint replacement and currently an intraarticular osteotomy is the only treatment option to prevent a TKA or a long-term impaired patient outcome [8].

A limitation of the study could be that the study size was relatively small and that subgroup analysis of i.e. poor outcome was limited. Moreover, with different types of malunion and surgical approaches, the population was heterogeneous. In this study, it was not possible to determine whether the leg axis or the intraarticular step-off was more important regarding patient-related outcomes. Thus, we need further studies to investigate whether the outcome is affected more by the reduced intraarticular step-off or the reduced postoperative leg malalignment. Also, postoperative radiographs to determine osteoarthritis at the latest follow-up were not available and this data could not be included in the analysis. Therefore, future studies are necessary to provide mid- to long-term follow-up to analyse the effect of intraarticular osteotomy on postoperative or posttraumatic osteoarthritis. Nevertheless, this is the first published case series on intraarticular osteotomy in patients with malunited TPF that determine risk factors of osteotomy failure.

## Conclusion

This study revealed that intraarticular osteotomy of malunited TPF lead to good clinical results with significant clinical and radiological improvement in most cases. After a follow-up of at least 2 years, an impaired patient outcome correlated with a limited range of motion. This study is the first analysis of risk factors of a poor outcome of intraarticular osteotomy.
